# Improving the leptospirosis disease burden assessment by including ambulatory patients from outpatient departments: a cross-sectional study

**DOI:** 10.12688/f1000research.26202.1

**Published:** 2020-09-14

**Authors:** Janith Warnasekara, Parami Aberathna, Geetha Nanayakkara, Joseph Vinetz, Suneth Agampodi

**Affiliations:** 1 Department of Community Medicine, Faculty of Medicine and Allied Sciences, Rajarata University of Sri Lanka, Saliyapura, Anuradhapura, 50008, Sri Lanka; 2 Department of Family Medicine, Faculty of Medicine and Allied Sciences, Rajarata University of Sri Lanka, Saliyapura, Anuradhapura, 50008, Sri Lanka; 3 Teaching Hospital Rathnapura, Rathnapura, Sri Lanka; 4 Section of Infectious Diseases, Department of Internal Medicine, School of Medicine, Yale University, New Haven, 208022, USA

**Keywords:** Leptospirosis, Sri Lanka, Outpatient department, Ambulatory care, OPD, burden, underestimation

## Abstract

**Background: **In Sri Lanka, the disease burden of leptospirosis is estimated based on a routine notification system, which is predominated by patients ill enough to be hospitalized. The notification system does not function well with ambulatory patients in outpatient departments (OPDs). The objective of this study was to determine the prevalence of leptospirosis in an OPD setting in a regional public hospital in Sri Lanka to provide further estimation of disease burden estimations

**Methods: **This study was conducted in the OPD of the Rathnapura Provincial General Hospital from August to September 2017. Suspected leptospirosis patients were recruited based on standardized criteria and tested using the microscopic agglutination test and quantitative polymerase chain reaction. The number of OPD patients was compared with the reported patient numbers with leptospirosis from the hospital during the same period as the denominator, and the 95% confidence interval was calculated for the proportions using Poisson distribution.

**Results: **During the study period, of 2,960 fever patients presenting to the OPD, 33 (1.1%) were suspected to have leptospirosis; 8/33 suspected (22.3%) cases were confirmed as being due to leptospirosis. There were 82 notifications of leptospirosis cases from hospital inpatients during the same period, none from the OPD. The total missing proportion from the surveillance system was 28.6% (95% CI, 19.4-40.4%). Among OPD patients, 12 (36.4%) had been given antibiotics from a primary care center prior to the OPD visit. No OPD patient was admitted to the hospital for inward care.

**Conclusions: **More than 25% of cases of leptospirosis were not identified because they were not sick enough to be admitted nor subjected to routine leptospirosis diagnostic testing. Antibiotics given without a specific, treatable diagnosis interferes with leptospirosis disease burden assessment. These data have public health implications if the sources of leptospirosis transmission are to be controlled.

## Introduction

Generally speaking, assessing the true burden of disease is required for proper health planning and resource allocation, including the control of transmissible diseases such as leptospirosis. Sri Lankan communicable disease burden estimates are usually done using routinely reported data in the surveillance system ^1^. Lack of actionable diagnostic tests and the diversity of clinical features leading to under-notification of leptospirosis are the major reasons for poor estimation of this disease, a leading cause of acute febrile illness in Sri Lanka ^2, 3^. A recently published systematic review has suggested a correction factor for hospitalized leptospirosis cases to more accurately estimate the burden of this disease. This study estimated the incidence of leptospirosis in Sri Lanka as 52.1 per 100,000 population ^3^. However, these estimations and corrections are made for hospitalized patients without considering outpatient departments (OPDs). It is estimated that approximately 5–15% of outpatients with undifferentiated febrile cases could be due to leptospirosis ^4, 5^, and undifferentiated febrile patients usually present to OPDs. Finally, these estimates have not been applied to assessing disability-adjusted life years, which is always a challenge for acute febrile illnesses. Therefore, prospective studies in outpatient setting are essential for estimating the burden of disease due to leptospirosis, which in turn is needed to justify investment in diagnostics and vaccine development.

Few studies have assessed leptospirosis in non-hospitalized patients with acute febrile illness. Biggs *et al.* highlighted the underestimation of leptospirosis due to non-inclusion of ambulatory patients for disease estimates in Tanzania ^6^. A study conducted in Vanuatu showed the importance of screening for leptospirosis among acute febrile illness patients presenting to OPDs during outbreaks, highlighting the need for improved awareness and diagnostic capacity, which are interrelated ^7^. In the Vanuatu study, 12 of 161 (7.4%) suspected patients were confirmed as having leptospirosis. However only 2 of 12 confirmed patients had criteria fulfilling the surveillance case definition, showing the inadequacy of the case definitions used ^7^. Another study conducted in Guadeloupe, Martinique (French territories in the Caribbean) suggested that the actual burden of leptospirosis could be 3 to 4 times higher than reported cases ^8^. A study conducted in Mozambique also provided supportive evidence for the importance of outpatient leptospirosis by estimating that as much as 10% of febrile patients attending ambulatory care could be attributed to leptospirosis ^9^. The purpose of the present study was to determine the prevalence of leptospirosis in an OPD setting in a regional public hospital in Sri Lanka to provide further estimation of disease burden estimations.

## Methods

### Setting

The study was conducted from August 2017 to September 2017 in the OPD of Rathnapura Provincial General Hospital (RPGH) as a part of larger clinico-epidemiological study. Previous data suggested that the Rathnapura district is one of four major districts affected by leptospirosis ^10^. At the time of the present study, the OPD had a separate desk for patients presenting with acute febrile illness. This was partly due to the massive epidemic of dengue ongoing during that period.

### Participants and data collection

Once the medical officer screened the patients for obvious foci of infection and after sending probable dengue patients for further investigation, a medical graduate awaiting an internship appointment screened the remaining acute undifferentiated fever patients. Clinically suspected patients were recruited as “possible” cases of leptospirosis, using a standardized, written surveillance case definition for Sri Lanka ^11^. In the meantime a survey was conducted among inward clinically confirmed leptospirosis patients of RPGH to assess the past treatment history.

Recruited patients were interviewed using a standardized, written clinical data checklist and a questionnaire ( *Extended data*). A blood sample of 4ml was taken, and 2ml was transferred to a plane tube and 2ml to an EDTA tube and stored in the microbiology laboratory of RPGH.

Samples were transported to the public health research laboratory of the Faculty of Medicine and Allied Sciences, Rajarata University of Sri Lanka. Testing for leptospirosis was done using the microscopic agglutination test (MAT) and quantitative polymerase chain reaction, as previously published in the study protocol ^12^.

Hospital notification data were obtained from the infection control unit at RPGH. The number of confirmed OPD patients was compared with the number of leptospirosis-confirmed hospitalized patients during the same period, and normalized to total patient populations. Care-seeking was compared with sample of hospitalized patients treated as leptospirosis by attending physicians.

### Data analysis

A SPSS trial version 23 was used for data analysis. A Poisson distribution was used to calculate the 95% confidence interval for the missing patient estimates from OPD.

### Ethical considerations

Ethical approval for this research was obtained from the Ethics Review Committee of the Faculty of Medicine and Allied Sciences, Rajarata University of Sri Lanka (No: ERC/2015/18). Written informed consent was obtained from all the patients for participation in the study.

## Results

**Figure 1.**
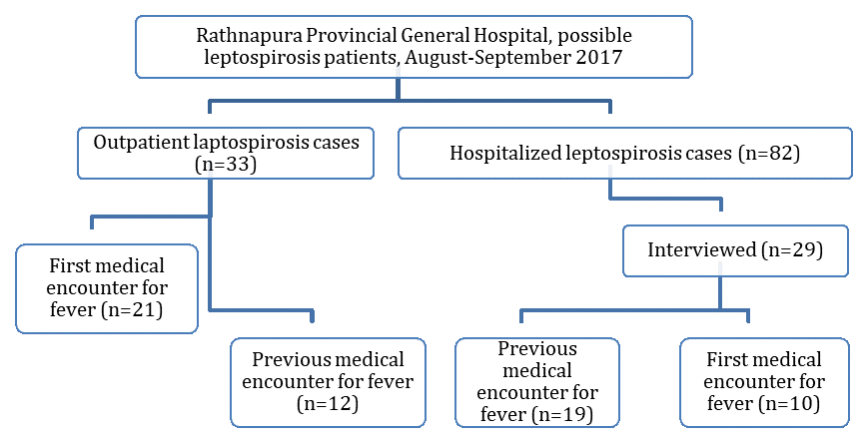
Flow chart of patient selection and diagnosis.

A total of 2,960 febrile patients were screened in the fever section of the OPD during the study period. Of these, 33 (1.1%) were clinically suspected leptospirosis patients and all were recruited for the present study ( Figure 1). These included 23 (69.7%) men and 10 (30.3%) women. The mean age was 46.5 years (SD 17.1). During the same period, RPGH made 82 notifications of possible cases of leptospirosis from hospitalized patients. The missing OPD patients from the notification accounted for 28.6% (95% CI 19.4-40.4) ( Table 1).

**Table 1.** Comparison of hospital reported cases with outpatient department cases.

Month	Total notifications	Notifiable cases from outpatient department	Percentage missing from surveillance system	95%-CI of percentage missing
August	31	11	26.1%	17.7-38.0
September	51	22	30.1%	21.0-42.8
Total	82	33	28.6%	19.4-40.4

Of 33 possible cases, 8 (24.2%) were laboratory confirmed as leptospirosis. One patient was categorized as “probable” with single MAT titre of 1/200 ^12^. Of the 33 cases selected, 12 (36.4%) had received treatment from a primary care centre prior to coming to the RPGH OPD. During the same period, we interviewed 29 hospitalized patients who were treated presumptively for leptospirosis. Of these, 19 (66.5%) reported that they were given treatment for fever from a primary care provider prior to hospital admission. However, none of these 19 visited the OPD of RPGH, confirming that the cases presented to OPD are really “missing” from the system.

## Discussion

In this preliminary study to evaluate the missing leptospirosis patient load in the surveillance system, we made three important observations: (1) almost one third of the patients presenting to the OPD of RPGH were missing from the notification system; (2) most of the patients (although we could say none, there might be admissions after the study period) presenting to the OPD were not hospitalized; (3) most of the hospitalized patients sought healthcare from primary care centres rather than from a tertiary care centre. Based on the OPD data, it clearly shows that 28.6% (95% CI 19.4-40.4) of the leptospirosis patients presenting to this tertiary centre were not included in the system. Nevertheless, statistical assumptions cannot be made for the primary care institution without proper studies conducted in local hospitals and private healthcare institutions. This study mainly focused on the cases presenting in an endemic setting and during an outbreak period. The missing numbers can neither be generalized to all areas of Sri Lanka nor for all the months of the year in the same area. Establishing well-functioning disease surveillance system in OPDs and primary care institutions is essential for proper disease burden estimates, not only for leptospirosis, but also for other notifiable diseases. There are various small scale studies that has been conducted to identify feasible methods for disease surveillance, such as incorporating smart phone technology which are being carried by hand by the treating physician ^13^. These feasibility studies need to be up scaled to identify the barriers and the feasible methods to implement the system. Well-planned studies covering outpatient, inpatient and private sector should be initiated for estimating the actual burden of diseases.

## Data availability

### Underlying data

Zenodo: OPD Lepto Data base - Clinical check List, http://doi.org/10.5281/zenodo.4013248 ^14^.

### Extended data

Zenodo: OPD Lepto Data base - Clinical check List, http://doi.org/10.5281/zenodo.4013248 ^14^.

This project contains the following extended data:

- Questionnaire OPD (1st Interview)
- Event calendar

Data are available under the terms of the Creative Commons Attribution 4.0 International license (CC-BY 4.0).
