# Supplementary material for: Improving the leptospirosis disease burden assessment by including ambulatory patients from outpatient departments: a cross-sectional study
Source: F1000Res. 2021 May 10;9:1129. Originally published 2020 Sep 14. [Version 2] doi: 10.12688/f1000research.26202.2 (PMC8145224; doi:10.12688/f1000research.26202.2)
Supplement: Peer review history

# Peer review: Reviewer response for version 2

**DOI:** 10.5256/f1000research.56435.r85002

**Published:** 2021-05-24

**Authors:** Chinthika P. Gunasekara

**Affiliations:** 1 Department of Microbiology, University of Sri Jayewardenepura, Nugegoda, Sri Lanka

I have gone through the revisions and the article is now acceptable for indexing.

Is the work clearly and accurately presented and does it cite the current literature?

Partly

If applicable, is the statistical analysis and its interpretation appropriate?

Yes

Are all the source data underlying the results available to ensure full reproducibility?

No

Is the study design appropriate and is the work technically sound?

Yes

Are the conclusions drawn adequately supported by the results?

Yes

Are sufficient details of methods and analysis provided to allow replication by others?

Partly

Reviewer Expertise:

NA

I confirm that I have read this submission and believe that I have an appropriate level of expertise to confirm that it is of an acceptable scientific standard.

# Peer review: Reviewer response for version 1

**DOI:** 10.5256/f1000research.28916.r82774

**Published:** 2021-04-19

**Authors:** Chinthika P. Gunasekara

**Affiliations:** 1 Department of Microbiology, University of Sri Jayewardenepura, Nugegoda, Sri Lanka

In this study the authors address an important aspect of leptospirosis burden in Sri Lanka that is a timely need. The main finding of the study that is conducted in a high prevalent district Rathnapura is that the true burden of leptospirosis is underestimated and about 28% cases coming to the OPD for treatment are not reported.

 I suggest that the authors include more recent references including data from the epidemiological unit as there are several publications done in Sri Lanka published more recently. 

 The supporting files are missing some components as the part C of the questionaire. Further the abstract results section mentions some data that is not described by way of table, additional data or described in the results section and this should be corrected to support the findings and conclusion. for example antibiotic history. 

 There are several spelling errors Eg : plane tube - plain tube. Overall I believe that the results section should be slightly expanded to support the conclusions and findings. This is an interesting paper and I recommend it for indexing subject to revision.

Is the work clearly and accurately presented and does it cite the current literature?

Partly

If applicable, is the statistical analysis and its interpretation appropriate?

Yes

Are all the source data underlying the results available to ensure full reproducibility?

No

Is the study design appropriate and is the work technically sound?

Yes

Are the conclusions drawn adequately supported by the results?

Yes

Are sufficient details of methods and analysis provided to allow replication by others?

Partly

Reviewer Expertise:

Leptospirosis, immune response

I confirm that I have read this submission and believe that I have an appropriate level of expertise to confirm that it is of an acceptable scientific standard, however I have significant reservations, as outlined above.

# Response

**Published:** 2021-04-24

**Authors:** Janith Warnasekara

**Affiliations:** Department of Community Medicine, Faculty of Medicine and Allie Sciences, Rajarata University of Sri Lanka, Sri Lanka

**Comment**

**I suggest that the authors include more recent references including data from the epidemiological unit as there are several publications done in Sri Lanka published more recently.**

**Reply**

 This was submitted as a **brief report** with a specific focus and the reference included were only directly related to the narrow focus. This was the reason for having a small results section. Extensive discussions on all Sri Lankan publications were done as a part of the larger study, which has included epidemiological unit data and data from all recent papers. (Published systematic review DOI: 10.1186/s12879-018-3655-y 6364467

**Comment**

**The supporting files are missing some components as the part C of the questionaire.**

****

**Reply**

 Yes. We understand that some of the phrases in questionnaire are mis leading. This study was conducted as a part of large clinical study related to leptospirosis. The Part C and further sections were for inward patients (not relevant to this brief report). We only applied Part A and B for OPD patients as the duration of interview with an OPD patient is less than the inward patients. To avoid misinterpretation, misleading parts were explained in the questionnaire. Corrected questionnaire is uploaded as extended data.

 Eg: Go to part C (Only the inward Patients)

**Comment**

**Further the abstract results section mentions some data that is not described by way of table, additional data or described in the results section and this should be corrected to support the findings and conclusion. for example antibiotic history.**

**Reply**

 First part of the results section of the abstract was explained in the figure one and the rest is in the table.

 We identify that 12 patients received antibiotics are mentioned as “antibiotics” in the abstract and as “treatment” in the main text.

 To avoid misinterpretation, we have changed the treatment as “antibiotic treatment” in the main text results section.

**Comment**

**There are several spelling errors Eg: plane tube - plain tube.**

**Reply**

 Spelling, grammar and formatting of the text are revised and corrected. All changes are mentioned as track changes in the manuscript.

**Comment**

**Overall I believe that the results section should be slightly expanded to support the conclusions and findings.**

**Reply**

 We humbly agree that the results section is small. As explained earlier, this is a brief report with very specific focus and we have included all the data relevant to this aim it this paper.

# Peer review: Reviewer response for version 1

**DOI:** 10.5256/f1000research.28916.r74988

**Published:** 2021-02-10

**Authors:** Georgies F Mgode

**Affiliations:** 1 Pest Management Centre, Sokoine University of Agriculture, Morogoro, Tanzania

The authors present important findings on leptospirosis from outpatients population. It is a well designed study and covered a large sample size consisting of 2,960 individuals with febrile illness. 

- In the methodology section: plane tube should be "plain tube".
- Further description of the microscopic agglutination test (MAT) is needed given that it is a core test for confirmation of leptospirosis. Information on Leptospira serovars used, age and whether was live or killed is much needed. 

Recommendation to establish a well-functioning disease surveillance system is important.

Is the work clearly and accurately presented and does it cite the current literature?

Yes

If applicable, is the statistical analysis and its interpretation appropriate?

Yes

Are all the source data underlying the results available to ensure full reproducibility?

Yes

Is the study design appropriate and is the work technically sound?

Yes

Are the conclusions drawn adequately supported by the results?

Yes

Are sufficient details of methods and analysis provided to allow replication by others?

Partly

Reviewer Expertise:

Rodent borne zoonotic diseases - leptospirosis and plague disease, tuberculosis and taxonomy

I confirm that I have read this submission and believe that I have an appropriate level of expertise to confirm that it is of an acceptable scientific standard.

# Response

**Published:** 2021-02-10

**Authors:** Janith Warnasekara

**Affiliations:** Department of Community Medicine, Faculty of Medicine and Allie Sciences, Rajarata University of Sri Lanka, Sri Lanka

Thank you very much for the response. I agree with the comments made by the reviewer. I would like to do the following changes in the manuscript.

1. Change the plane into plain in methodology
2. Add a supplementary file of full MAT protocol 

Thank you very much
